# Genome-wide association study of abdominal MRI-measured visceral fat: The multiethnic cohort adiposity phenotype study

**DOI:** 10.1371/journal.pone.0279932

**Published:** 2023-01-06

**Authors:** Samantha A. Streicher, Unhee Lim, S. Lani Park, Yuqing Li, Xin Sheng, Victor Hom, Lucy Xia, Loreall Pooler, John Shepherd, Lenora W. M. Loo, Thomas Ernst, Steven Buchthal, Adrian A. Franke, Maarit Tiirikainen, Lynne R. Wilkens, Christopher A. Haiman, Daniel O. Stram, Iona Cheng, Loïc Le Marchand

**Affiliations:** 1 University of Hawaii Cancer Center, University of Hawaii at Mānoa, Honolulu, Hawaii, United States of America; 2 Department of Epidemiology and Biostatistics, University of California–San Francisco, San Francisco, California, United States of America; 3 Center for Genetic Epidemiology, Department of Preventive Medicine, Keck School of Medicine, University of Southern California, Los Angeles, California, United States of America; 4 University of Maryland School of Medicine, Baltimore, Maryland, United States of America; Wake Forest School of Medicine, UNITED STATES

## Abstract

Few studies have explored the genetic underpinnings of intra-abdominal visceral fat deposition, which varies substantially by sex and race/ethnicity. Among 1,787 participants in the Multiethnic Cohort (MEC)-Adiposity Phenotype Study (MEC-APS), we conducted a genome-wide association study (GWAS) of the percent visceral adiposity tissue (VAT) area out of the overall abdominal area, averaged across L1-L5 (%VAT), measured by abdominal magnetic resonance imaging (MRI). A genome-wide significant signal was found on chromosome 2q14.3 in the sex-combined GWAS (lead variant rs79837492: Beta per effect allele = -4.76; P = 2.62 × 10^−8^) and in the male-only GWAS (lead variant rs2968545: (Beta = -6.50; P = 1.09 × 10^−9^), and one suggestive variant was found at 13q12.11 in the female-only GWAS (rs79926925: Beta = 6.95; P = 8.15 × 10^−8^). The negatively associated variants were most common in European Americans (T allele of rs79837492; 5%) and African Americans (C allele of rs2968545; 5%) and not observed in Japanese Americans, whereas the positively associated variant was most common in Japanese Americans (C allele of rs79926925, 5%), which was all consistent with the racial/ethnic %VAT differences. In a validation step among UK Biobank participants (N = 23,699 of mainly British and Irish ancestry) with MRI-based VAT volume, both rs79837492 (Beta = -0.026, P = 0.019) and rs2968545 (Beta = -0.028, P = 0.010) were significantly associated in men only (n = 11,524). In the MEC-APS, the association between rs79926925 and plasma sex hormone binding globulin levels reached statistical significance in females, but not in males, with adjustment for total adiposity (Beta = -0.24; P = 0.028), on the log scale. Rs79837492 and rs2968545 are located in intron 5 of *CNTNAP5*, and rs79926925, in an intergenic region between *GJB6* and *CRYL1*. These novel findings differing by sex and racial/ethnic group warrant replication in additional diverse studies with direct visceral fat measurements.

## Introduction

Excess body fatness is an established risk factor for type 2 diabetes, cardiovascular disease, and multiple common cancers [1, 2]. Abdominal visceral adipose tissue (VAT) has been shown to be more metabolically active compared to fat stored in other areas of the body, such as subcutaneous adipose tissue (SAT) [3, 4]. Recent studies have shown strong associations between VAT and type 2 diabetes, cardiovascular disease, and breast cancer [3, 5, 6]. VAT has greater metabolic activity and direct access to the portal circulation, which promotes an abnormal metabolic profile (i.e. circulating levels of high insulin, leptin, sex steroids, adiponectin, C-reactive protein) [7–10].

It is well established that the absolute or relative size of VAT differs substantially by sex and race/ethnicity [11, 12]. In the Multiethnic Cohort-Adiposity Phenotype Study (MEC-APS) (N = 1,861), we found that men had substantially more magnetic resonance imaging (MRI)-measured abdominal VAT compared to women, even after adjusting for age, height, and total adiposity [12]. Differences in sex-specific fat accumulation become evident at the onset of puberty with women preferentially accumulating fat in the gluteofemoral region and men accumulating more fat as visceral fat in the abdominal region [13]. In women, when estrogen levels decrease during their menopausal transition, fat redistributes away from the gluteofemoral region towards more visceral fat [13]. In addition, results from an analysis of a large extended pedigree from a genetic isolate in the Netherlands showed that genes account for significantly more variance for waist circumference, hip circumference, and waist to hip ratio in women (mean age = 47.5 years) than in men (mean age = 48.5) [14]: Different genes were found contributing to the variance of the waist to hip ratio in men compared to women, and common genes accounted for a larger magnitude of the variance for waist and hip circumference in women than men [14].

In the MEC-APS, the amount of MRI-measured VAT also significantly differed among the five race/ethnicities (African American, European American, Japanese American, Latino, and Native Hawaiian), even after adjusting for age, height, and total adiposity [12]. Japanese Americans had the largest VAT area in both males and females (234 cm^2^ and 176 cm^2^, respectively) and African Americans had the smallest VAT area in both males and females (161 cm^2^ and 102 cm^2^, respectively), adjusted for total adiposity [12]. In another study in the MEC, for similar body mass index (BMI), Japanese American women, compared to White women, had a significantly higher MRI-measured VAT area out of the abdominal area across L4-L5 (23.9% vs. 18.5%, respectively), after adjusting for age and total adiposity [15].

Accounting for possible sex-differences in genetic susceptibility variants, several studies have conducted sex-stratified genome-wide association studies (GWAS) of computed tomography (CT)- or MRI-measured VAT [16–20]. Two genome-wide significant variants (rs2842895 and rs2185405) were associated with abdominal VAT together in males and females; another two variants (rs11118316 and rs7374732) were associated with the abdominal VAT to SAT ratio together in males and females; three novel variants (rs1659258, rs10060123, and rs17104731) were associated with abdominal VAT in females-only; and two variants (rs12657394 and rs1002945) were associated with abdominal VAT in males-only [16–20]. An additional study using 396,220 UK Biobank participants predicted VAT mass from dual energy X-ray absorptiometry (DXA) and found 101 variants associated with predicted VAT mass at genome-wide significance in males and females, and one additional variant in males [21]. However, these studies have mostly been in individuals of European ancestry.

Recognizing the need to further elucidate the genetic determinants of VAT variation by sex and race/ethnicity based on accurate measurements, we performed a sex-combined and sex-specific GWAS study of visceral adiposity, evaluated by MRI in the MEC-APS. We, then, examined variants that were genome-wide significant (P<5x10^-8^) and suggestive (P<10^−7^) for association with obesity-related biomarkers in MEC-APS and for replication with VAT in the UK Biobank data.

## Methods

### The MEC-APS

The MEC is an ongoing prospective study in Hawaii and Los Angeles that was established to examine the association of lifestyle and genetic risk factors with cancer and other chronic diseases. In 1993–1996, 96,810 men and 118,441 women between 45 and 75 years of age were recruited. Participants are mainly from five main racial/ethnic groups (African American, European American, Japanese American, Latino, Native Hawaiian) [22]. The MEC-APS was conducted in 2013–2016 among a subset of MEC participants to identify predictors of body fat distribution and risk factors for obesity-related cancers, as described previously [12]. Briefly, the MEC-APS is a cross-sectional study that recruited 1,861 healthy, nonsmoking men and women between 60 and 77 years of age with body mass index (BMI) between 17.1–46.2 kg/m^2^. MEC participants were selected for the study using a stratified sampling by sex, race/ethnicity, and six BMI categories. Exclusion criteria included reported BMI outside the range of 18.5–40 kg/m^2^, smoking in the past 2 years, soft or metal body implants or amputation, insulin or thyroid medications, and serious medical conditions (e.g., dialysis, chronic hepatitis, previous cancer diagnosis). Study participants underwent an abdominal MRI and a whole-body dual energy X-ray absorptiometry (DXA) scan, provided a blood sample after an overnight fast, completed a self-administered questionnaire, and underwent anthropometric measurements [12]. All MEC-APS participants provided written informed consent and the study was approved by the institutional review boards (IRBs) at the University of Hawaii (UH) (CHS-#17200), University of Southern California (USC) (#HS-12-00623), and University of California, San Francisco (UCSF) (#17–23399) in agreement with the 1975 Helsinki Declaration. Seventy-four participants were excluded because of missing visceral fat value based on an invalid scan due to implants, motion artifacts, or presence of a visceral mass, leaving 1,787 MEC-APS participants in the final study population.

### Anthropometric and body composition assessment

Trained technicians obtained measurements of height, weight, circumferences of the waist and hip, and chest depth [12]. 3T MRI scanners (Siemens TIM Trio at UH and General Electric HDx at USC) were used. An abdominal scan was acquired to quantify VAT areas (square centimeters) at 4 intervertebral segments of the intra-abdominal cavity (L1–L2, L2–L3, L3–L4, L4–L5) using an axial gradient-echo sequence with breath holds [12]. The average VAT across the segments L1-L5 was used in analysis. Whole-body composition, including total fat mass and muscle mass, was determined by a DXA scan (Hologic Discovery A fan-beam densitometer at UH and USC, Bedford, MA) [12]. Extensive details regarding the imaging protocol, as well as quality control calibration and estimation of VAT and SAT area were previously published [12].

### Genotyping, quality control, and imputation

Genotyping and imputation for the MEC-APS participants have been described previously [23]. Briefly, DNA extraction from buffy coat was performed using the Qiagen QIAMP DNA kit (Qiagen Inc., Valencia, CA). DNA samples were genotyped on the Illumina expanded multi-ethnic genotyping array (MEGA^EX^) platform, which provides a large coverage of variants across the genome for diverse ancestral populations [24]. Variants were removed if they had a call rate <95%, replicate concordance <100% based on 39 QC replicate samples, or poor clustering after visual inspection. Prior to imputation, monomorphic variants, variants with a call rate <98%, variants with estimated MAF (minor allele frequency) that deviated by ≥20% in comparison to the corresponding ancestral group in the 1000 Genomes Project Phase 3, discordance in reported vs. genotyped sex, and insertions/deletions that were not included in the Haplotype Reference Consortium (HRC), were removed. From an initial 2,036,060 genotyped variants, 1,417,570 were available for imputation. Phasing using Eagle v2.4 and genotype imputation using Minimac v4 were performed on the University of Michigan Imputation Server with the HRC vr1.1 2016 reference panel [25, 26]. After genotype imputation for MEC-APS participants, variants with an imputation quality score of < 0.4, multiallelic variants, variants with MAF <0.01, or monomorphic variants, were excluded from all subsequent analyses. In total, 9,542,479 genotyped and imputed variants remained after post-imputation filtering. Principal components for ancestry adjustment were calculated with 16,621 post-quality control genotyped race/ethnic specific pruned single nucleotide polymorphisms using EIGENSOFT v7 [27]. A quantile–quantile plot of GWAS P-values indicated appropriate control of type I error for the total population, males, and females, with a genomic inflation (λ) value of 0.98, 0.98, and 0.96, respectively (S1A–S1C Fig).

### Obesity-related biomarkers

Selected blood biomarkers were assayed because of their reported associations with obesity-caused metabolic, hormonal, and inflammation dysfunctions [5]. Fasting blood samples were collected at the time of body composition measurement, processed into components, and stored at -80°C [5]. Plasma or serum concentrations were determined for circulating levels of high density lipoprotein (HDL) (mg/dL) (N = 1,822), total cholesterol (mg/dL) (N = 1,823), glucose (mg/dL) (N = 1,821), C-reactive protein (CRP) (mg/dL) (N = 1,823), insulin (microU/mL) (N = 1,823), sex-hormone binding globulin (SHBG) (nmol/L) (N = 1,816), triglycerides (mg/dL) (N = 1,823), and alanine aminotransferase (ALT) (U/L) (N = 1,823) [5]. HOMA-IR (N = 1,821) and HOMA-beta (N = 1,810) were derived from fasting glucose and insulin values [5, 28, 29], and low-density lipoprotein (LDL; N = 1,817) cholesterol was derived from the Friedewald equation using total and HDL cholesterol values and a valid range of triglyceride concentrations [30].

### UK Biobank

The UK Biobank participants, genotyping, imputation, and imaging have been described in detail previously [31–33]. Briefly, the UK Biobank recruited over 500,000 individuals, aged 40–69 years, mainly of white British ancestry from across the UK during 2006–2010 [31]. Participants were interviewed about lifestyle and disease history and underwent a physical examination that included measurements on weight, height, and waist and hip circumference [31]. Genotyping for UK Biobank participants was done with two custom genotyping arrays, UK BiLEVE and Axiom [32]. The UK10K and 1000 Genomes Phase 3 reference panels were used as reference panels for imputation [25, 32, 34]. Between 2014 and 2020 a subset of 43,521 UK Biobank participants underwent MRI imaging. VAT volume (data field: 22407), measured by summing the VAT area across images, was quantified by abdominal MRI in a subset of 25,103 participants using a Siemens 1.5 T MAGNETOM Aera scanner (Siemen, Erlangen, Germany) with the dual-echo Dixon Vibe protocol covering neck to knees [33, 35]. This analysis of data from the UK Biobank was performed under UK Biobank application #16447.

### Statistical analysis

Descriptive characteristics were examined in the overall study population, in males, and in females, by race/ethnicity (African American, European American, Japanese American, Latino, Native Hawaiian) using SAS v.9.4.

The median percent VAT area out of the overall abdominal area, averaged across L1-L5 (%VAT) presented a greater contrast across the five race/ethnic groups compared to absolute VAT area, averaged over L1-L5 (using Mass-Whitney U test; P = 2.3x10^-31^ vs. P = 2.3x10^-14^, respectively); therefore, %VAT was used for the GWAS outcome. Variants (as imputed dosages) were tested for associations among sex-combined (N = 1,787) MEC-APS participants using linear regression of %VAT on SNPs using additive genetic models, adjusted for age, sex, and principal components 1–4. Sex-specific (male n = 878, female n = 909) GWAS were also performed using linear regressions of %VAT, adjusted for age and sex-specific principal components 1–4 with additive genetic models. SNP associations were considered statistically significant at the genome-wide significance threshold of P<5x10^-8^, and as suggestive at P<10^−7^. To evaluate independent variant effects, conditional analysis was then conducted that included variants from the same chromosome signal with P<10^−7^. Since %VAT was correlated, albeit weakly, with total fat mass (r = 0.12), its associations with the independent lead variants were rerun with additional adjustment for total adiposity. All analyses were done in PLINK v2.0. The %VAT outcome was not transformed because the non-transformed error residuals were normally distributed and a quantile-quantile plot of GWAS P-values of variant associations indicated appropriate control of type I errors with genomic inflation (λ) value for GWAS was close to 1 (S1A–S1C Fig) [36].

Lead variants associated with %VAT from the sex-combined and sex-specific GWAS were also assessed for relationships within each MEC-APS race/ethnic group (African American n = 301, European American n = 401, Japanese American n = 428, Latino n = 372, Native Hawaiian n = 285) using linear regression and adjusted for age, sex (for variants associated with %VAT in sex-combined data), and race/ethnic-specific principal components in PLINK v2.0.

Okinawan Americans were genetically distinguished from mainland Japanese Americans among 428 Japanese Americans in the MEC-APS using principal component analysis. The principal component 1 vs. principal component 2 plot allowed for visualization of the spread of Japanese Americans (S2 Fig). Identification of Okinawan Americans was based on previous principal component analysis plots that identified Okinawans and where subject clusters thinned [37]. Cross-check of participants in the Okinawan cluster with Okinawan last names indicated that the majority of the last names in the Okinawan cluster were of Okinawan origin. There were 72 genetically identified Okinawan Americans, 27 part-Okinawan and part-mainland Japanese Americans, and 333 mainland Japanese Americans (S2 Fig). Medians and P-values of % VAT between Okinawan Americans and mainland Japanese were calculated using SAS v.9.4.

Lead genetic variants with %VAT were also examined for association with obesity-related blood biomarkers (HDL, LDL, total cholesterol, glucose, insulin, HOMA-beta, HOMA-IR, CRP, SHBG, triglycerides, and ALT) among over 1,800 MEC-APS participants [5] using linear regression of each log-transformed biomarker on each lead genetic variant adjusted for age, sex (for variants associated with %VAT in sex-combined data), total fat mass, and principal components 1–4 (see above in *Obesity-related biomarkers* for exact number of participants analyzed for each biomarker) using R v3.6.1. The same lead genetic variants were also assessed for replication in the UK Biobank using linear regression models of log-transformed VAT volume adjusted for BMI, age, sex, and principal components 1–4 using PLINK v.2.0.

## Results

The GWAS study population consisted of 1,787 MEC-APS participants (Table 1). The stratified recruitment into MEC-APS resulted in similar numbers of study participants across sex-race/ethnicity-BMI categories. The median overall age at clinic visit was 69.8 years. Across all race/ethnicities males had higher %VAT compared to females (Table 1). Overall, and in males and females, Japanese Americans (overall = 26.6%, male = 30.11%, female = 22.8%) had the highest % VAT, followed by Latinos (overall = 25.6%, males = 30.10%, females = 22.4%), Native Hawaiians (overall = 23.3%, males = 27.0%, females = 20.8%), and European Americans (overall = 28.8%, males = 26.8%, females = 18.7%). African Americans had the lowest %VAT overall (19.0%) and in males (30.11%) and females (16.6%) (Table 1).

**Table 1:** Descriptive characteristics of MEC-APS subjects with valid visceral fat area and genotyping values, overall and by race/ethnicity (N = 1787)^a^.

	Overall	African American	European American	Japanese American	Latino	Native Hawaiian
**All, N (%)**	1787	301(16.7)	401 (22.4)	428 (24.0)	372 (20.8)	285 (15.9)
Age at clinic visit, years	69.1 (67.1, 71.3)	69.8 (67.9, 72.0)	68.6 (67.1, 70.9)	68.7 (66.8, 70.5)	69.7 (67.4, 72.1)	68.4 (66.5, 71.1)
Sex, n (%)						
Male	878 (49.1)	126 (41.9)	207 (51.6)	227 (53.0)	187 (50.2)	131 (46.0)
Female	909 (50.9)	174 (58.1)	194 (48.3)	201 (47.0)	185(49.8)	154 (54.0)
Visceral fat to abdominal area ratio	23.5 (17.9, 29.9)	19.0 (14.9, 24.5)	21.7 (16.5, 28.2)	26.6 (20.2, 32.3)	25.6 (20.7, 26.4)	23.3 (18.1, 28.9)
Subcutaneous fat to abdominal area ratio	32.5 (25.9, 40.7)	40.1 (32.5, 47.4)	28.8 (23.2, 38.1)	28.1 (24.0, 35.1)	34.9 (27.1, 42.9)	34.5 (27.5, 40.3)
Visceral fat area (L1-L5), cm^2^	157.4 (105.6, 215.0)	135.5 (94.6, 193.4)	141.2 (93.9, 204.7)	159.3 (104.4, 210.8)	188.9 (132.6, 248.1)	156.5 (107.8, 215.2)
Subcutaneous fat area (L1-L5), cm2	211.6 (154.3, 282.4)	272.5 (205.8, 359.5)	191.1 (136.0, 267.8)	166.3 (130.0, 217.1	236.4 (180.5, 312.7)	220.3 (172.0, 286.6)
Abdominal area (L1-L5), cm^2^	658.8 (555.9, 770.1)	700.0 (587.6, 823.3)	649.8 (552.6, 759.7)	579.5 (502.9, 686.6)	708.2 (599.3, 812.6)	665.8 (566.9, 771.9)
Visceral fat area/subcutaneous fat area	0.74 (0.48, 1.15)	0.49 (0.34, 0.61)	0.71 (0.49, 0.88)	0.96 (0.62, 1.36)	0.80 (0.53, 1.22)	0.71 (0.50, 1.05)
Total fat mass, kg	24.2 (18.8, 29.9)	29.0 (24.0, 36.2)	22.3 (17.5, 27.6)	19.8 (16.3, 24.1)	27.9 (23.2, 33.2)	23.2 (19.5, 28.0)
Body mass index, kg/m^2^	27.3 (24.4, 30.8)	28.9 (25.3, 32.2)	26.4 (23.5, 29.8)	25.9 (23.0, 28.9)	28.5 (25.7, 32.2)	28.2 (25.2, 31.7)
**Men, n (%)**	878 (49.1)	126 (14.3)	207 (23.6)	227 (25.8)	187 (21.3)	131 (14.9)
Age at clinic visit, years	69.2 (67.1, 71.4)	70.2 (67.8, 72.3)	68.3 (66.9, 70.8)	68.7 (66.8, 70.5)	69.7 (67.4, 72.3)	69.2 (66.8, 71.4)
Visceral fat to abdominal area ratio	28.1 (21.9, 33.2)	24.4 (19.8, 29.7)	26.8 (18.4, 32.2)	30.1 1 (24, 34.1)	30.1 0 (26, 35.4)	27 (20.8, 32.1)
Subcutaneous fat to abdominal area ratio	26.2 (22.3, 30.7)	31.2 (26.3, 36.9)	24.4 (21.1, 28.5)	24.1 (20.7, 27)	27.8 (23.9, 32.4)	27.4 (23.2, 31.5)
Visceral fat area (L1-L5), cm^2^	198.6 (137.2, 254.4)	181 (134.1, 232.1)	187.9 (114.4, 248.7)	186.8 (130.2, 243.8)	236.8 (182.4, 283.2)	190.2 (133, 248.3)
Subcutaneous fat area (L1-L5), cm2	178.4 (136.6, 230.9)	216.6 (169.4, 295.1)	167.9 (130.5, 211.1)	148.2 (113.7, 182.3)	203.1 (153.1, 256.7)	195.4 (145, 242.3)
Abdominal area (L1-L5), cm^2^	688.2 (591.9, 798.5)	717.3 (642.5, 846.8)	683 (601.9, 777.5)	620.2 (519.3, 707.5)	742.8 (651.7, 842.9)	703.2 (598.4, 813.3)
Visceral fat area/subcutaneous fat area	1.1 (0.83, 1.5)	0.8 (0.6, 1.1)	1.1 (0.8, 1.5)	1.3 (1, 1.7)	1.2 (0.9, 1.6)	1.1 (0.8, 1.3)
Total fat mass, kg	22.1 (17.5, 27.1)	26.9 (22.7, 32.6)	20.5 (16.3, 25.5)	18.5 (15.1, 22.2)	26.1 (21.8, 30.5)	21.5 (17.9, 26.4)
Body mass index, kg/m^2^	27.1 (24.8, 30.3)	28.3 (25.8, 31.1)	26.5 (24.1, 29.5)	26 (23.1, 28.8)	28.2 (26, 31.3)	28.2 (25.7, 31.4)
**Women, n (%)**	909 (50.9)	175 (19.3)	154 (16.9)	201 (22.1)	185 (20.3)	194 (21.3)
Age at clinic visit, years	69.0 (67.1, 71.2)	69.5 (67.9, 71.7)	69 (67.1, 70.9)	68.7 (66.8, 70.6)	69.5 (67.3, 71.9)	67.9 (66.1, 70.8)
Visceral fat to abdominal area ratio	20.1 (16.0, 24.5)	16.6 (13, 19.8)	18.7 (14.9, 22.7)	22.8 (18, 27.8)	22.4 (18.5, 25.2)	20.8 (16.8, 24.8)
Subcutaneous fat to abdominal area ratio	39.5 (33.9, 45.4)	45.5 (40.4, 50.7)	37.8 (30.1, 44.2)	35 (29.8, 39.1)	42.4 (37.3, 48)	38.9 (35.1, 44.1)
Visceral fat area (L1-L5), cm^2^	128.9 (91.5, 170.9)	114.1 (82.9, 150.1)	118 (76.7, 162.6)	128.5 (92, 176.6)	145.7 (111.2, 190.3)	140.2 (97.1, 180.6)
Subcutaneous fat area (L1-L5), cm2	246.7 (189.4 316.1)	298.5 (229.4, 401.2)	239.6 (154.1, 294.1)	192.8 (154.9, 240.2)	287.3 (222.7, 366.5)	248.8 (206.9, 310.4)
Abdominal area (L1-L5), cm^2^	622.9 (531.0, 736.9)	679.4 (556.8, 793.2)	642.6 (540.6, 730.2)	559 (483.1, 639.3)	677.1 (578.2, 785.1)	600.8 (488, 720.6)
Visceral fat area/subcutaneous fat area	0.51 (0.39, 0.68)	0.4 (0.3, 0.5)	0.5 (0.4, 0.7)	0.6 0.5, 0.9)	0.5 (0.4, 0.7)	0.5 (0.4, 0.7)
Total fat mass, kg	26.4 (21.2, 32.9)	33.4 (26.6, 39)	25 (18.9, 30.4)	21.8 (18.4, 25.1)	29.7 (26, 36.4)	25.5 (21.1, 30.7)
Body mass index, kg/m^2^	27.4 (24.1, 31.4)	29.1 (25.1, 33.6)	26.2 (22.8, 30.1)	25.5 (22.7, 28.6)	28.7 (25.4, 32.9)	28.3 (24.6, 32.4)

^a^Count (percentage) of categorical variables and median (interquartile range) of continuous variables are presented across race/ethnicity of MEC-APS participants.

**Fig 1:**
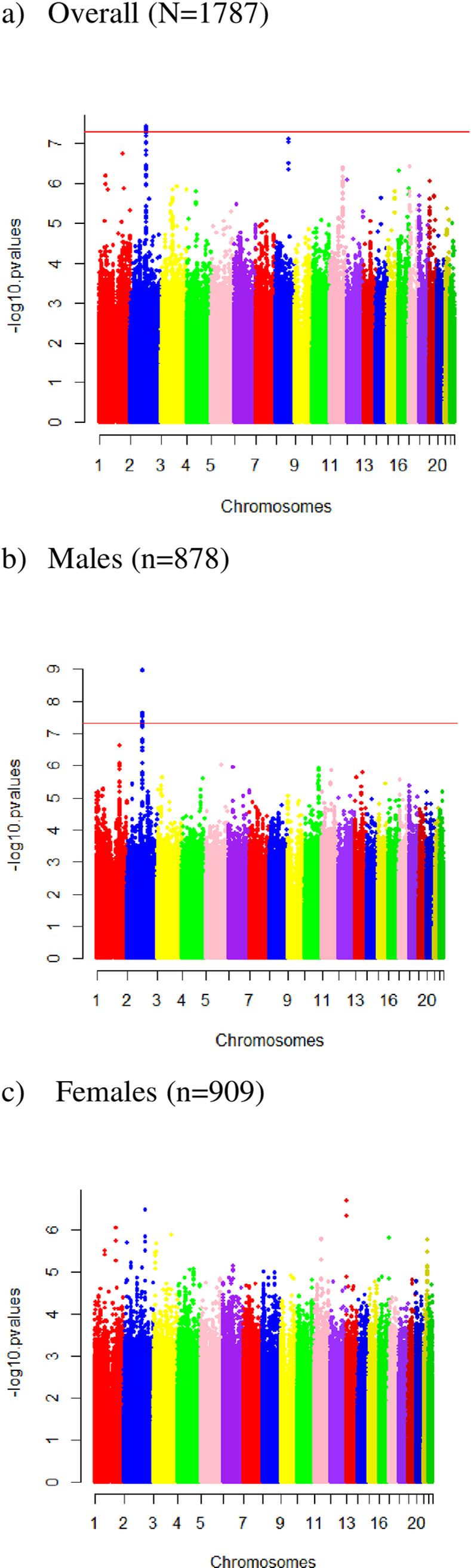
Manhattan plots of SNP P-values from the visceral fat to abdominal area ratio genome-wide association study in the Multiethnic Cohort-Adiposity Phenotype Study (MEC-APS). The Y-axis shows the negative base ten logarithm of the P-values and the X-axis shows the chromosomes. The genome-wide significance threshold, P<5x10^-8^, is shown in red: a) Overall (N = 17,87), b) Males (n = 878), c) Females (n = 909).

**Fig 2:**
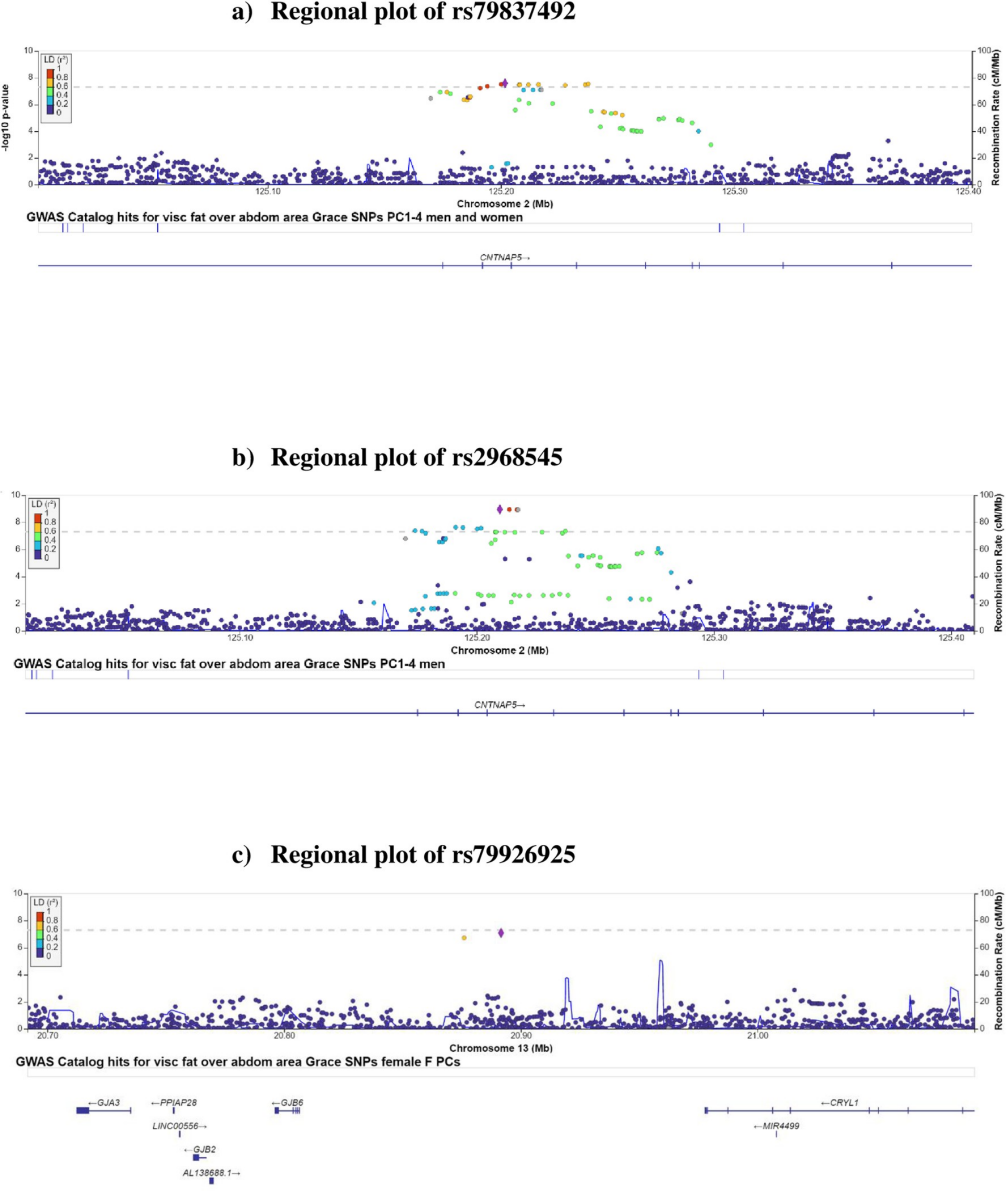
Regional plots of SNP P-values in a +/-200 kb window around rs79837429, rs2968545, and rs79926925. The X-axis shows the chromosome and physical location (Mb), the left Y-axis shows the negative base ten logarithm of the P-values, and the right Y-axis shows recombination activity (cM/Mb) as a blue line. Positions, recombination rates, and gene annotations are according to NCBI’s build 37 (hg 19) and the 1000 Genomes Project Phase 3 multiethnic data set: a) Regional plot of rs79837429, b) Regional plot of rs2968545, c) Regional plot of rs79926925.

In the sex-combined MEC-APS GWAS, there was a signal on chromosome 2q14.3, located in intron 5 of *CNTNAP5*, with 11 genome-wide significant variants and five suggestive variants (P<10^−7^), associated with %VAT (Figs 1A and 2A and Table 2A). The most significant association (rs79837492) was the lead variant in a conditional analysis that included these 16 variants with P<10^−7^. This 16-variant signal on chromosome 2q14.3 is located in intron 5 of *CNTNAP5* (Contactin Associated Protein Family Member 5). The T allele of the lead variant, rs79837492, was associated with a mean decrease of 4.76 in %VAT per effect allele (P = 2.62 x 10^−8^), independent of age, sex, and principal components (Table 2A). With additional adjustment for total fat mass, rs79837492 was associated with %VAT at P = 2.50 x 10^−6^ (Beta per effect allele = -3.74) (S1 Table). The variant T allele of rs79837492 was most common in European Americans (5%), present at lower frequency in African Americans (3%), Latinos (2%) and Native Hawaiians (1%) and not observed in Japanese Americans (Table 3A). The most significant association across race/ethnicities between rs79837492 and %VAT was in African Americans (Beta = -6.15; P = 1.0 × 10^−4^) with consistent effect estimates and directions of associations in the other non-monomorphic populations (Table 3A).

**Table 2:** Genetic variants associated with the ratio of visceral fat to abdominal area in the MEC-APS (P<10^−7^).

**a) Sex-combined (N = 1787)**									
**Variant**	**Chr** ^**c**^	**Position** ^**d**^	**Imputed Info score**	**Ref Allele**	**Effect Allele**	**EAF** ^**e**^	**Beta**	**SE**	**P-value**
**rs79837492** ^**a,b**^	2	125201730	0.96	C	T	0.022	-4.76	0.85	2.62x10^-8^
**rs75293929**	2	125237347	0.98	A	G	0.022	-4.7	0.86	2.94x10^-8^
**rs77575492**	2	125200134	0.96	A	G	0.022	-4.7	0.85	3.12x10^-8^
**rs74805958**	2	125216045	0.99	T	C	0.022	-4.66	0.84	3.25x10^-8^
**rs111548554**	2	125211775	0.99	A	T	0.022	-4.66	0.84	3.34x10^-8^
**rs76381612**	2	125236095	0.98	A	C	0.022	-4.65	0.84	3.37x10^-8^
**rs76377414**	2	125208103	1.00	A	C	0.022	-4.66	0.84	3.43x10^-8^
**rs76651976**	2	125208020	1.00	A	G	0.022	-4.66	0.84	3.43x10^-8^
**rs75297301**	2	125207690	genotyped	T	C	0.022	-4.66	0.84	3.45x10^-8^
**rs111545964**	2	125227517	0.98	G	A	0.024	-4.5	0.81	3.70x10^-8^
**rs79269543**	2	125194156	0.95	C	T	0.023	-4.63	0.84	4.50x10^-8^
**rs78658619**	2	125191084	0.93	T	C	0.023	-4.61	0.85	6.14x10^-8^
**rs2420864**	2	125217386	0.99	C	T	0.033	-4.66	0.68	8.10x10^-8^
**rs77575492**	2	125216924	0.99	A	G	0.033	-4.66	0.68	8.11x10^-8^
**rs2420858**	2	125213719	0.99	G	T	0.033	-4.66	0.68	8.24x10^-8^
**rs2968545**	2	125209666	0.99	A	C	0.033	-4.66	0.68	8.38x10^-8^
**b) Male-only (n = 878)**									
**Variant**	**Chr** ^**c**^	**Position** ^**d**^	**Imputed Info score**	**Ref Allele**	**Effect Allele**	**EAF** ^**e**^	**Beta**	**SE**	**P-value**
**rs2968545** ^**a,b**^	2	125209666	0.99	A	C	0.033	-6.5	1.06	1.09x10^-9^
**rs2420858**	2	125213719	0.99	G	T	0.033	-6.65	1.06	1.13x10^-9^
**rs77575492**	2	125216924	0.99	A	G	0.033	-6.65	1.06	1.16x10^-9^
**rs2420864**	2	125217386	0.99	C	T	0.033	-6.65	1.06	1.17x10^-9^
**rs78658619**	2	125191084	0.93	C	T	0.023	-7.2	1.28	2.33x10^-8^
**rs79269543**	2	125194156	0.95	C	T	0.024	-7.2	1.26	2.43x10^-8^
**rs79837492**	2	125201730	0.96	C	T	0.023	-7.2	1.29	2.70x10^-8^
**rs77575492**	2	125200134	0.96	A	G	0.023	-7.1	1.27	3.06x10^-8^
**rs113164486**	2	125173963	0.87	G	A	0.024	-7.36	1.33	4.10x10^-8^
**rs75293929** ^**a,c**^	2	125237347	0.98	A	G	0.022	-7.03	1.28	4.49x10^-8^
**rs76524201**	2	125176877	0.88	A	G	0.024	-7.3	1.32	4.55x10^-8^
**rs75297301**	2	125207690	genotyped	T	C	0.023	-7.16	1.26	5.08x10^-8^
**rs76377414**	2	125208103	1	A	C	0.023	-6.9	1.26	5.10x10^-8^
**rs76651976**	2	125208020	1.00	A	G	0.023	-6.9	1.26	5.10x10^-8^
**rs111545964**	2	125227517	0.98	G	A	0.025	-6.7	1.23	5.20x10^-8^
**rs111548554**	2	125211775	0.99	A	T	0.023	-6.8	1.26	5.27x10^-8^
**rs74805958**	2	125216045	0.99	T	C	0.023	-6.9	1.26	5.51x10^-8^
**rs76381612**	2	125236095	0.98	A	C	0.023	-6.9	1.26	6.16x10^-8^
**rs77919433**	2	125178343	0.88	C	G	0.024	-7.22	1.26	6.28x10^-8^
**b) Female-only (n = 909)**									
**SNP**	**Chr** ^**c**^	**Position** ^**d**^	**Imputed Info score**	**Ref Allele**	**Effect Allele**	**EAF** ^**e**^	**Beta**	**SE**	**P-value**
**rs79926925** ^**a,b**^	13	20891618	0.99	T	C	0.013	6.95	1.29	8.15x10^-8^

^a^Adjusted for age, sex, and overall principal components 1–4. ^b^For rs79837492, there were approximately 79 T alleles in the overall MEC-APS population. ^c^Chr, chromosome. ^d^Position according to NCBI build37. ^e^EAF, Effect allele frequency.

^a^Adjusted for age, sex, and overall principal components 1–4. ^b^For rs2968545, there were approximately 58 C alleles in self-reported males. ^c^Chr, chromosome. ^d^Position according to NCBI build37. ^e^EAF, Effect allele frequency.

^a^Adjusted for age, sex, and overall principal components 1–4. ^b^For rs79926925, there were approximately 24 C alleles in self-reported females. ^c^Chr, chromosome. ^d^Position according to NCBI build37. ^e^EAF, Effect allele frequency.

**Table 3:** Genetic variants associated with percent visceral fat in the MEC-APS (P<10^07^), by race/ethnicity.

**a) Sex-combined (N = 1787)** ^a^																					
		**African American (n = 301)**				**European American (n = 401)**				**Japanese American (n = 428)**				**Latino (n = 372)**				**Native Hawaiian (n = 285)**			
**Variant**	**Chr** ^b^	**EAF** ^c^	**Beta**	**SE**	**P**	**EAF** ^c^	**Beta**	**SE**	**P**	**EAF** ^c^	**Beta**	**SE**	**P**	**EAF** ^c^	**Beta**	**SE**	**P**	**EAF** ^c^	**Beta**	**SE**	**P**
**rs79837492**	2	0.026	-6.15	1.56	0.00010	0.049	-4.76	1.26	0.00019	-	-	-	-	0.02	-2.62	1.79	0.14	0.011	-7.68	2.97	0.0101
**rs75293929**	2	0.030	-5.29	1.45	0.00031	0.047	-4.84	1.29	0.00021	-	-	-	-	0.02	-2.91	1.79	0.10	0.011	-7.73	2.88	0.0078
**rs77575492**	2	0.028	-5.91	1.50	0.00010	0.049	-4.77	1.27	0.00019	-	-	-	-	0.02	-2.56	1.79	0.15	0.011	-7.60	2.98	0.0112
**rs74805958**	2	0.029	-5.26	1.45	0.00034	0.048	-4.72	1.28	0.00026	-	-	-	-	0.02	-2.92	1.79	0.10	0.010	-7.84	2.89	0.0071
**rs111548554**	2	0.029	-5.24	1.45	0.00036	0.048	-4.72	1.28	0.00026	-	-	-	-	0.02	-2.92	1.79	0.10	0.010	-7.86	2.90	0.0072
**rs76381612**	2	0.030	-5.28	1.45	0.00031	0.048	-4.72	1.28	0.00026	-	-	-	-	0.02	-2.92	1.79	0.10	0.011	-7.71	2.86	0.0075
**rs76377414**	2	0.029	-5.21	1.45	0.00038	0.048	-4.72	1.28	0.00026	-	-	-	-	0.02	-2.92	1.79	0.10	0.010	-7.87	2.90	0.0072
**rs76651976**	2	0.029	-5.21	1.45	0.00038	0.048	-4.72	1.28	0.00026	-	-	-	-	0.02	-2.92	1.79	0.10	0.010	-7.87	2.90	0.0072
**rs75297301**	2	0.029	-5.21	1.45	0.00039	0.048	-4.72	1.28	0.00026	-	-	-	-	0.02	-2.92	1.79	0.10	0.010	-7.87	2.90	0.0072
**rs111545964**	2	0.039	-4.67	1.28	0.00030	0.048	-4.72	1.28	0.00025	-	-	-	-	0.02	-2.63	1.78	0.14	0.011	-7.77	2.88	0.0073
**rs79269543**	2	0.029	-5.93	1.49	0.00009	0.049	-4.80	1.27	0.00018	-	-	-	-	0.02	-2.35	1.78	0.19	0.012	-7.33	3.00	0.0152
**rs78658619**	2	0.029	-5.97	1.51	0.00009	0.049	-4.82	1.27	0.00017	-	-	-	-	0.02	-2.26	1.79	0.21	0.012	-7.10	3.00	0.0188
**rs2420864**	2	0.094	-3.13	0.83	0.00020	0.049	-4.32	1.27	0.00072	-	-	-	-	0.02	-2.19	1.63	0.18	0.011	-7.84	2.89	0.0071
**rs77575492**	2	0.094	-3.12	0.83	0.00020	0.049	-4.32	1.27	0.00072	-	-	-	-	0.02	-2.19	1.63	0.18	0.011	-7.84	2.89	0.0071
**rs2420858**	2	0.094	-3.12	0.83	0.00020	0.049	-4.32	1.27	0.00072	-	-	-	-	0.02	-2.19	1.63	0.18	0.011	-7.86	2.90	0.0071
**rs2968545**	2	0.094	-3.12	0.83	0.00021	0.049	-4.32	1.27	0.00073	-	-	-	-	0.02	-2.19	1.63	0.18	0.011	-7.86	2.90	0.0072
**b) Male-only (n = 878)** ^a^																					
		**African American (n = 126)**				**European American (n = 207)**				**Japanese American (n = 216)**				**Latino (n = 187)**				**Native Hawaiian (n = 142)**			
**Variant**	**Chr** ^b^	**EAF** ^c^	**Beta**	**SE**	**P**	**EAF** ^c^	**Beta**	**SE**	**P**	**EAF** ^c^	**Beta**	**SE**	**P**	**EAF** ^**c**^	**Beta**	**SE**	**P**	**EAF** ^c^	**Beta**	**SE**	**P**
**rs2968545** ^**a**^ ^**,**^ ^**b**^	2	0.113	-5.93	1.27	8.6x10^-6^	0.052	-9.10	1.93	4.69x10^-6^	-	-	-	-	0.018	3.01	2.81	0.28	0.0035	-11.32	7.72	0.15
**rs2420858**	2	0.114	-5.91	1.27	9.1x10^-6^	0.052	-9.11	1.93	4.65x10^-6^	-	-	-	-	0.018	3.01	2.81	0.29	0.0035	-11.35	7.72	0.14
**rs77575492**	2	0.114	-5.90	1.27	9.5x10^-6^	0.052	-9.11	1.93	4.62x10^-6^	-	-	-	-	0.018	3.01	2.81	0.29	0.0036	-11.37	7.72	0.14
**rs2420864**	2	0.114	-5.89	1.27	9.6x10^-6^	0.052	-9.11	1.93	4.62x10^-6^	-	-	-	-	0.018	3.01	2.81	0.29	0.0036	-11.37	7.72	0.14
**rs78658619**	2	0.047	-7.26	2.08	0.0006809	0.052	-9.12	1.94	4.90x10^-6^	-	-	-	-	0.017	-3.80	2.93	0.20	0.0042	-11.27	7.70	0.15
**rs79269543**	2	0.048	-7.12	2.05	0.0006981	0.052	-9.11	1.94	4.85x10^-6^	-	-	-	-	0.018	-3.65	2.91	0.21	0.0041	-11.32	7.72	0.15
**rs79837492**	2	0.043	-7.70	2.18	0.0005728	0.052	-9.11	1.94	4.77x10^-6^	-	-	-	-	0.018	-3.37	2.86	0.24	0.0038	-11.35	7.73	0.14
**rs77575492**	2	0.047	-7.11	2.07	0.0008152	0.052	-9.11	1.94	4.79x10^-6^	-	-	-	-	0.018	-3.44	2.87	0.23	0.0039	-11.36	7.73	0.14
**rs113164486**	2	0.043	-8.20	2.27	0.0004323	0.052	-8.75	1.98	1.66x10^-6^	-	-	-	-	0.018	-4.12	3.14	0.19	0.0043	-11.18	7.73	0.15
**rs75293929**	2	0.047	-6.67	2.06	0.0015253	0.049	-9.44	1.97	3.14x10^-6^	-	-	-	-	0.018	-3.02	2.81	0.28	0.0034	-12.00	8.10	0.14
**rs76524201**	2	0.044	-8.06	2.24	0.0004630	0.052	-8.69	1.98	1.81x10^-6^	-	-	-	-	0.018	-4.07	3.10	0.19	0.0041	-11.40	7.74	0.14
**rs75297301**	2	0.046	-6.86	2.06	0.0011554	0.052	-9.10	1.93	4.73x10^-6^	-	-	-	-	0.018	-3.02	2.81	0.28	0.0035	-11.32	7.72	0.15
**rs76377414**	2	0.046	-6.86	2.06	0.0011629	0.052	-9.10	1.93	4.72x10^-6^	-	-	-	-	0.018	-3.02	2.81	0.28	0.0035	-11.32	7.72	0.15
**rs76651976**	2	0.046	-6.86	2.06	0.0011607	0.052	-9.10	1.93	4.73x10^-6^	-	-	-	-	0.018	-3.02	2.81	0.28	0.0035	-11.32	7.72	0.15
**rs111545964**	2	0.057	-6.33	1.89	0.0011162	0.052	-9.11	1.93	4.59x10^-6^	-	-	-	-	0.018	-2.78	2.81	0.32	0.0036	-11.39	7.73	0.14
**rs111548554**	2	0.046	-6.83	2.06	0.0012208	0.052	-9.10	1.93	4.70x10^-6^	-	-	-	-	0.018	-3.02	2.81	0.28	0.0035	-11.34	7.72	0.14
**rs74805958**	2	0.046	-6.78	2.06	0.0013164	0.052	-9.11	1.93	4.65x10^-6^	-	-	-	-	0.018	-3.02	2.81	0.28	0.0035	-11.37	7.72	0.14
**rs76381612**	2	0.047	-6.66	2.06	0.0015556	0.052	-9.11	1.93	4.54x10^-6^	-	-	-	-	0.018	-3.02	2.81	0.28	0.0036	-11.43	7.73	0.14
**rs77919433**	2	0.045	-8.01	2.22	0.0004510	0.052	-8.63	2.00	2.4x10^-5^	-	-	-	-	0.018	-4.04	3.09	0.19	0.0041	-11.36	7.73	0.14
**b) Female-only (n = 909)** ^a^																					
		**African American (n = 175)**				**European American (n = 154)**				**Japanese American (n = 201)**				**Latino (n = 185)**				**Native Hawaiian (n = 194)**			
**Variant**	**Chr** ^b^	**EAF** ^c^	**Beta**	**SE**	**P**	**EAF** ^c^	**Beta**	**SE**	**P**	**EAF** ^**c**^	**Beta**	**SE**	**P**	**EAF** ^c^	**Beta**	**SE**	**P**	**EAF** ^c^	**Beta**	**SE**	**P**
**rs79926925**	13	-	-	-	-	-	-	-	-	0.048	6.37	1.21	5.65x10^-5^	-	-	-	-	0.012	6.68	3.26	0.042

^a^Adjusted for age, sex, and race/ethnic specific principal components 1–4.

^b^Chr, chromosome.

^c^EAF, Effect allele frequency.

In the male-specific GWAS of %VAT, the signal on chromosome 2q14.3 gained magnitude and strength: all 16 significant or suggestive variants from the sex-combined GWAS were significant in men, with two additional significant variants (rs113164486 and rs76524201) and an additional suggestive variant (rs77919433) associated with %VAT (Figs 1B and 2B and Table 2B). The most significant association (rs2968545) was the lead variant in a conditional analysis that included the 19 variants from the chromosome 2q14.3 signal with P<10^−7^ in males. The variant C allele of the lead variant, rs2968545, was associated with a mean decrease of 6.5 per effect allele in %VAT (P = 1.09 x 10^−9^), independent of age and principal components (Table 2B). With additional adjustment for total fat mass, rs2968545 was associated with the %VAT at P = 2.50 x 10^−6^ (Beta = -3.74) (S1 Table). In males, the C allele of rs2968545 was most common in African Americans (11%), less common in European Americans (5%), present at low frequency in Latinos (1.8%) and Native Hawaiians (0.03%), and not observed in Japanese Americans (Table 3B). The most significant association across race/ethnicities among men between rs2968545 and %VAT was in European Americans (Beta = -9.10; P = 4.69 × 10^−6^) with consistent effect estimates and directions of associations in the other non-monomorphic populations (Table 3B).

None of the 19 variants identified in the male GWAS on chromosome 2 were associated with %VAT in females, but all betas were in the same direction (S2A and S3A Tables). In the female MEC-APS GWAS of %VAT, there was a suggestive association with rs79926925 and %VAT on chromosome 13q12.11 in an intergenic region between *GJB6* (Gap Junction Beta 6 or Connexin 30) and *CRYL1 (*crystallin lambda 1). The variant C allele of rs79926925 on chromosome 13q12.11 was associated with a mean increase of 6.95 in %VAT (P = 8.15x10^-8^) independent of age and principal components (Fig 1C and Table 2C). With additional adjustment for total fat mass, the rs79926925 association was attenuated only slightly at P = 3.93 x 10^−7^ (Beta per allele = 6.15) (S1 Table). In females, the C allele of rs79926925 was most frequent in Japanese Americans (5%), present at low frequency in Native Hawaiians (1.2%), and not observed in African Americans, European Americans, or Latinos (Table 3C). The most significant association across race/ethnicities among women between rs79926925 and %VAT was in Japanese Americans (Beta = 6.73; P = 5.65 × 10^−5^) with consistent effect estimates and directions of associations in Native Hawaiians (Table 3C). Rs79926925 was not associated with %VAT in males (Beta = 1.58, P = 0.35) (S2B and S3B Tables), but the beta was in the same direction. Okinawan Americans were found to have a higher frequency (7%) compared to mainland Japanese (3.5%) for the variant C allele of rs79926926. In women, (Okinawan Americans n = 23 and mainland Japanese Americans n = 54) the magnitude of association with the C allele of rs79926926 was similar in Okinawan Americans (Beta = 4.1, P = 0.50) and in mainland Japanese Americans (Beta = 5.5, P = 0.043), adjusted for age and sex-specific principal components. Consistent with their greater allele frequency, Okinawan American women also had significantly more %VAT (median = 25.1) compared to mainland Japanese Americans (median = 22.0) (P = 0.0073).

Several biomarkers were significantly associated with the lead variant (rs79837492) in expected directions from the sex-combined GWAS (CRP, insulin, SHBG, and triglycerides) and the lead variant (rs2968545) from the male-only GWAS (glucose, HOMA-IR, insulin, and triglycerides), at P<0.05; however, after adjusting for total adiposity, none of the biomarker associations remained statistically significant (Table 4A). Rs2968545 was not significantly associated with any biomarkers in females (S4A Table). The lead variant from the female-only GWAS, rs79926925, was associated with SHBG: The C allele of rs79926925 was associated with a 0.21-fold decrease (Beta = -0.24; P = 0.028) in geometric mean for SHBG in females (Table 4C). Rs79926925 was not significantly associated with any biomarkers in males (S4B Table).

**Table 4:** The association between lead significant and suggestive variants associated with percent visceral fat and obesity-related biomarkers in the MEC-APS, adjusted for total fat mass.

**a) Sex-combined**					
**Variant**	**Biomarker**	**N**	**Beta** ^**a,b**^	**SE**	**P-value**
rs79837492	HDL (mg/dL)	1813	-0.015	0.053	0.77
	LDL (mg/dL)	1808	-0.056	0.050	0.26
	Total Cholesterol (mg/dL)	1814	-0.033	0.034	0.33
	Glucose (mg/dL)	1812	-0.033	0.029	0.25
	HOMA-beta (%)	1801	-0.037	0.103	0.72
	HOMA-IR	1814	-0.033	0.086	0.70
	CRP (mg/L)	1814	-0.318	0.214	0.14
	Insulin (microU/mL)	1814	-0.055	0.077	0.50
	SHBG (nmol/L)	1807	0.086	0.061	0.16
	Triglycerides (mg/dL)	1814	-0.082	0.056	0.15
	ALT (U/L)	1814	-0.067	0.061	0.27
**b) Males**					
**Variant**	**Biomarker**	**N**	**Beta** ^**a,b**^	**SE**	**P-value**
rs2968545	HDL (mg/dL)	897	-0.003	0.060	0.96
	LDL (mg/dL)	894	-0.037	0.060	0.53
	Total Cholesterol (mg/dL)	898	-0.033	0.041	0.43
	Glucose (mg/dL)	896	-0.045	0.032	0.16
	HOMA-beta (%)	894	-0.102	0.117	0.38
	HOMA-IR	896	-0.119	0.095	0.21
	CRP (mg/L)	898	-0.209	0.251	0.40
	Insulin (microU/mL)	898	-0.126	0.090	0.16
	SHBG (nmol/L)	895	0.056	0.063	0.38
	Triglycerides (mg/dL)	898	-0.077	0.065	0.24
	ALT (U/L)	898	-0.030	0.069	0.66
**c) Females**					
**Variant**	**Biomarker**	**N**	**Beta** ^**b**^	**SE**	**P-value**
rs79926925	HDL (mg/dL)	916	-0.026	0.091	0.78
	LDL (mg/dL)	914	-0.055	0.079	0.49
	Total Cholesterol (mg/dL)	916	-0.047	0.053	0.37
	Glucose (mg/dL)	916	0.055	0.49	0.26
	HOMA-beta (%)	907	0.049	0.17	0.77
	HOMA-IR	916	0.23	0.15	0.12
	CRP (mg/L)	916	0.47	0.35	0.18
	Insulin (microU/mL)	916	0.19	0.13	0.13
	SHBG (nmol/L)	912	-0.24	0.11	0.028
	Triglycerides (mg/dL)	916	0.057	0.092	0.54
	ALT (U/L)	916	-0.006	0.10	0.96

^a^Adjusted for age, sex, principal components 1–4, and total fat mass (kg). ^b g^Log unit change per allele increase.

^a^Adjusted for age, sex specific principal components 1–4, and total fat mass (kg). ^b g^Log unit change per allele increase.

The UK Biobank VAT volume data set was comprised of 23,784 participants of white British or Irish ancestry, 211 of Asian ancestry (Indian, Pakistani, or Bangladeshi), 135 of African or Caribbean ancestry, and 973 of other or mixed ancestry. In UK Biobank participants, both rs79837492 (sex-combined Beta = -0.007, P = 0.38; male-only Beta = -0.026, P = 0.019, female-only Beta = 0.011, P = 0.34) and rs2968545 (sex-combined Beta = -0.008, P = 0.30; male-only Beta = -0.028, P = 0.010, female-only Beta = 0.011, P = 0.37) were significantly associated in males, but not in females, with log-transformed MRI-measured VAT volume adjusted for body mass index (BMI), age, sex, and principal components 1–4 (Table 5). The frequency of the effect allele was higher in the UK Biobank compared to MEC-APS for both rs79837492 ((Effect allele frequency) EAF = 0.051 vs. EAF = 0.022, respectively) and rs2968545 (EAF = 0.052 vs. EAF = 0.033, respectively) (Table 5). Consistent with MEC-APS, the allele frequency for males and females was similar for both rs79837492 (male EAF = 0.050 and female EAF = 0.050) and rs2968545 (male EAF = 0.052 and female EAF = 0.050) (Table 5). The variant C allele of rs79926925 was not observed in participants with MRI-measured VAT volume in the UK Biobank.

**Table 5:** The association between lead significant variants associated with visceral fat volume (cm^3^) in the UK Biobank^a^.

		Sex-combined (N = 23,699)				Male (n = 11,524)				Female (n = 12,175)			
Variant	Chr	EAF	Beta^b^	SE	P	EAF	Beta^b^	SE	P	EAF	Beta^b^	SE	P
**rs79837492**	2	0.051	-0.007	0.007	0.38	0.050	-0.026	0.011	0.019	0.050	0.011	0.012	0.34
**rs2968545**	2	0.052	-0.008	0.008	0.30	0.051	-0.028	0.011	0.010	0.050	0.011	0.012	0.37

^a^adjusted for body mass index, age, sex, and principal components 1–4.

^b^log unit change per allele increase.

Four published GWAS of VAT have detected genome-wide significant associations [16, 18–20] (S5 Table). In addition, there have been two GWAS that found genome-wide significant variants using the UK Biobank data, one that explored predicted VAT mass derived from DXA measurements and one MedRxiv preprint that examined MRI-measured VAT mass [21] (S5 Table). There was evidence of replication in MEC-APS (P<0.05) for seven variants of the 115 previously identified genome-wide significant variants: rs113658831 (P = 0.031), rs2949785 (P = 0.010), rs56398417 (P = 0.031), rs10962547 (P = 0.018), rs7942037 (P = 0.0053), rs3764002 (P = 0.041), rs4239060 (P = 0.020), and rs1329254 (P = 0.029) (S5 Table).

## Discussion

In our GWAS of the %VAT in a racially/ethnically diverse population, we observed a genome-wide significant signal on chromosome 2q14.3 (lead variant rs79837492) in the sex-combined analysis, the same genome-wide significant signal on chromosome 2q14.3 (lead variant rs2968545) in the male-only GWAS, and a suggestive variant (rs79926925) on chromosome 13q12.11 in the female-only GWAS. The genome-wide significant signal on chromosome 2q14.3 is located in intron 5 of *CNTNAP5*. The suggestive variant, rs79926925 on chromosome 13q12.11 is located in an intergenic region between *GJB6* and *CRYL1*. Both rs79837492 and rs2968545 were associated with a decrease in mean %VAT; rs79837492 was significantly associated with CPR, SHBG, and triglycerides, and rs2968545 was significantly associated with glucose, HOMA-IR, insulin, and triglycerides at P = 0.05 in MEC-APS without adjustment for total adiposity, but neither variant was significantly associated with the obesity-related biomarkers after total adiposity adjustment. The association for both rs79837492 and rs2968545 with %VAT replicated at P = 0.05 in the UK Biobank. Rs79926925 was associated with an increase in mean %VAT and a significant decrease in mean SHBG blood levels, and was more common in Okinawan Americans than mainland Japanese Americans.

Since rs79837492 and rs2968545 are intronic and rs79926925 is in an intergenic region, these variants may function through mechanisms that regulate transcriptional activity. There were no eQTLs for either variant when querying the GTEx Portal [38]. *CNTNAP5* is a large gene (~1M bases) that is part of the Caspr family, a family of genes that are involved in cell contacts and communication in the nervous system [39]. Familial deletions, GWAS, and Multivariate and Collapsing (CMC) burden tests, in *CNTNAP5* have mainly found associations with atypical neurodevelopment and intellectual disability [40–43]. However, a genetic variant (rs314944) just downstream of *CNTNAP5* was found to be moderately negatively associated with abdominal visceral adiposity in a Korean study (P = 4.25 x 10^−5^), and whole blood gene expression of *CNTNAP5* was reported to be reduced after bariatric surgery in patients with type 2 diabetes [19, 44]. Rs79926925 was only present in Japanese American and Native Hawaiian MEC-APS participants, and is located in an intergenic region between *GJB6* and *CRYL1*. *CRYL1* is part of the uronate cycle, which functions as an alternative glucose metabolic pathway, catabolizing 5% of daily glucose [45]. *CRYL1* requires NAD(H) as a coenzyme to catalyze the dehydrogenation of L-gulonate into dehydro-L-gulonate [45]. The variant rs7989332 in *CRYL1* was found to interact with rs6455128 in *KHDRBS2* (KH domain containing, RNA binding, signal transduction associated 2) and be protective against Alzheimer’s Disease [46]. More recently, bioinformatics analysis has shown that *CRYL1* is a shared susceptibility gene between late-stage hepatocellular carcinoma and high HBA1c, and that the amount of *CRYL1* is inversely related to tumor stage [47]. Additionally, experiments in rabbits show that *CRYL1* is downregulated in skeletal muscle cells of obese, but not normal weight animals [48].

Recent multiethnic GWAS have found it beneficial, in a multiethnic population, to conduct a joint GWAS, compared to meta-analyzing separate GWAS stratified by race/ethnicity [49, 50]. Wojcik and colleges (2019) showed that a joint GWAS increases power compared to a meta-analysis approach, while maintaining the type I error rate [49]. In MEC, the first four principal components are able to differentiate the five different race/ethnicities (African American, European American, Japanese American, Latino, and Native Hawaiian). Wang and colleagues (2010) showed that African American, European American, and Japanese American MEC participants separate on principal components 1 and 2, Latinos separate on principal component 3, and Native Hawaiians separate on principal component 4 [51]. The first four principal components only adjusts for some intra-population structure (e.g. mainland Japanese vs. Okinawan) associations between significant or suggestive genetic variants. However, %VAT was also examined in race/ethnicity specific models that can account for intra-population structure by adjusting for race/ethnicity specific principal components. Related individuals (20 pairs of first degree relatives) were not removed from the GWAS because relatedness was limited to pairs of individuals who did not form related subpopulations within the data set. When relatedness does not lead to the creation of subpopulations in a data set, retaining related individuals does not cause long-range LD leading to misleading associations or loss of power [27, 52, 53].

Among Japanese Americans in the MEC, this is the first time Okinawan Americans have been distinguished from mainland Japanese Americans. Historically, Okinawa had the highest life expectancy from birth among all Japanese prefectures with low rates of chronic disease [54]. Today Okinawa have some of the highest chronic disease rates in Japan, believed to be due in part to a Westernized lifestyle/diet introduced by the US military presence since World War II [55]. Our novel findings in Okinawan Americans may imply unidentified gene-environment factors that warrant further investigation.

Rs79837492 and rs2968545 were genome-wide significant (P = 5x10^-8^) in the sex-combined and male-only MEC-APS GWAS and significant at P = 0.05 in the male-only UK Biobank replication. Neither rs79837492 nor rs2968545 were genome-wide significant in the female-only MEC-APS GWAS or the UK Biobank female-only replication. While these lead MEC-APS variants replicated in the UK Biobank, there are some study differences that could explain study-depended strengths of association. First, MEC-APS examined VAT as a proportion of abdominal area, whereas the UK Biobank provided data on VAT volume as an absolute measure, and second, because the EAF for rs79837492 and rs2968545 differ between studies, it is also possible that the white and black participants’ ancestry may differ between studies. The variant rs79926925 was not found in UK Biobank participants with abdominal MRI imaging, most likely because rs79926925 was not observed in whites and only present in Japanese Americans and Native Hawaiians in MEC-APS, and the UK Biobank MRI imaging study selected mainly for participants of white British and Irish ancestry [35].

Of the 115 previously identified genome-wide significant variants with VAT outcomes, only seven replicated in MEC-APS (P<0.05) [16, 18–20]. Reasons for non-replication of 108 previously identified genome-wide significant variants may be attributed to different racial/ethnic compositions of the study population, dissimilar VAT outcomes (i.e. absolute VAT, VAT adjusted for BMI, VAT/SAT, and predicted VAT), and use of CT or DXA (VAT measured by DXA only approximates VAT measured by the gold-standard MRI) for abdominal imaging [56].

To our knowledge, this is the first study to conduct GWAS for %VAT and one of the few studies to examine the genetics of visceral fat in a multiethnic population. Additional strengths of our study include the use of the MEGA^EX^ genotyping array, which provides comprehensive coverage of variants for a multiethnic population and the use of MRI to measure abdominal adiposity, which is the gold standard method for measuring visceral fat. The study population, however, was modest in size (N = 1,787) and, thus, statistical power to detect weak to moderate effects was limited. A power analysis with 1,787 subjects (population mean = 24.0, standard deviation = 8.3) shows that a GWAS would have > 80% power to detect a variant with 2% change in mean %VAT with a MAF > 0.15 (at P = 5x10^-8^) [57].

In summary, we found a significant signal on chromosome 2q14.3 in the sex-combined (lead variant rs79837492) and male-only (lead variant rs2968545) GWAS of %VAT, and one suggestive variant (rs79926925) in the female-only GWAS of %VAT in MEC-APS. The negatively associated lead variants (rs79837492 and rs2968545) were most common in European Americans and African Americans and the positively associated lead variant (rs79926925) was most common in Japanese Americans: this was all consistent with the racial/ethnic %VAT differences. The variant allele of rs79926925, associated with a mean increase in %VAT in women, also showed an association with decreased blood levels of SHBG. These findings should be considered as preliminary and they require replication in larger studies using gold-standard methods (MRI or CT) to measure visceral fat.

## Supporting information

S1 Fig Q-Q plot of SNP P-values from the ratio of visceral fat to abdominal area GWAS, all MEC-APS participants, and by male and female MEC-APS participants. The Y-axis shows the negative base ten logarithm of the observed p-values and the X-axis shows the negative base ten logarithm of the expected p-values.
(DOCX)

S2 Fig Principal component plot 2 vs. 1 for 432 Japanese Americans in the Multiethnic Cohort-Adiposity Phenotye Study (MEC-APS). The vertical line demarcates separation between Japanese Americans and part-Japanese Americans. The top horizontal line demarcates separation between Okinawan Americans and part-Okinawan & part-mainland Japanese Americans and the bottom horiontal line demarcates separation between part-Okinawan & part-mainland Japanese Americans and mainland-Japanese Americans.
(DOCX)

S1 Table Lead variants (P<10–7) from the visceral fat to abdominal area ratio GWAS further adjusted for total fat mass (kg). (XLSX)

S2 Table Genetic variants with P<10−7 from the self-reported male and female GWAS on the visceral fat to abdominal area ratio, in the other sex: a) Female (n = 909), b) Male (n = 878).
(XLSX)

S3 Table Genetic variants with P<10^−7^ from the self-reported male and female GWAS on the visceral fat to abdominal area ratio, in the other sex by race/ethnicity. (XLSX)

S4 Table The association between lead significant and suggestive variants associated with the visceral fat to abdominal area ratio and obesity-related biomarkers in the MEC-APS, in the other sex. (XLSX)

S5 Table Replication in the Multiethnic Cohort-Adiposity Phenotype Study (MEC-APS) of previously novel significant (P<5x10^-8^) variant associations with visceral adipose tissue and the ratio of visceral adipose tissue to subcutaneous adipose tissue. (XLSX)
