# Supplementary material for: Genome-wide association study of abdominal MRI-measured visceral fat: The multiethnic cohort adiposity phenotype study
Source: PLoS One. 2023 Jan 6;18(1):e0279932. doi: 10.1371/journal.pone.0279932 (PMC9821421; doi:10.1371/journal.pone.0279932)
Supplement: S1 Fig — The Y-axis shows the negative base ten logarithm of the observed p-values and the X-axis shows the negative base ten logarithm of the expected p-values. (DOCX) [file pone.0279932.s001.docx]

**S1 Fig. Q-Q plot of SNP P-values from the ratio of visceral fat to abdominal area GWAS, all MEC-APS participants, and by male and female MEC-APS participants. The Y-axis shows the negative base ten logarithm of the observed p-values and the X-axis shows the negative base ten logarithm of the expected p-values.**

1. **All MEC-APS participants, genomic inflation λ=0.98**

**
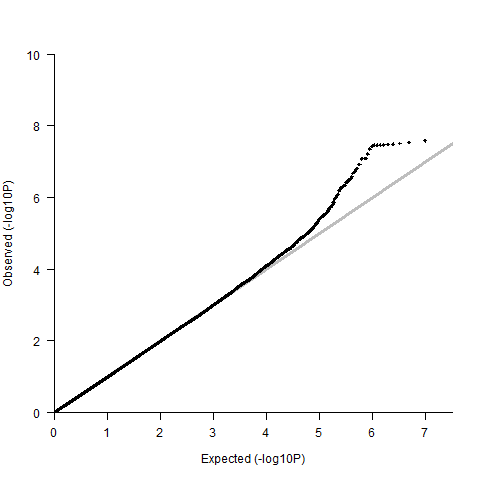
**

1. **Male MEC-APS participants, genomic inflation λ=0.98**

**
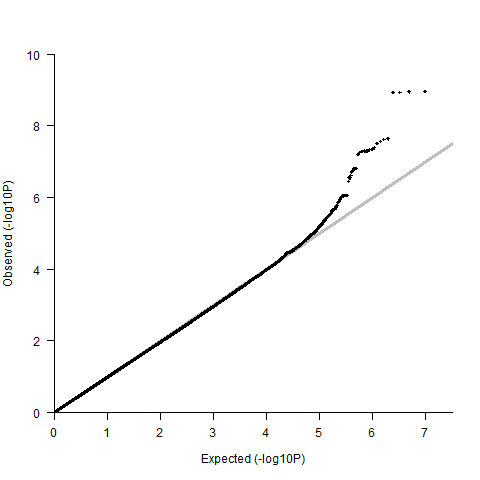
**

1. **Female MEC-APS participants, genomic inflation λ=0.96**

**
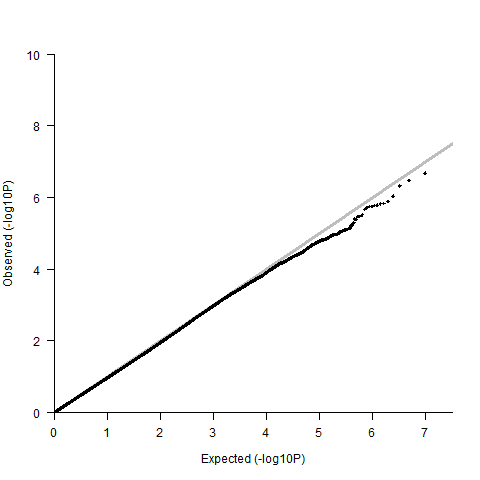
**
