# Supplementary material for: Genome-wide association study of abdominal MRI-measured visceral fat: The multiethnic cohort adiposity phenotype study
Source: PLoS One. 2023 Jan 6;18(1):e0279932. doi: 10.1371/journal.pone.0279932 (PMC9821421; doi:10.1371/journal.pone.0279932)

**S2 Fig. Principal Component plot 2 vs. 1 for 432 Japanese Americans in the Multiethnic Cohort-Adiposity Phenotye Study (MEC-APS). The vertical line demarcates separation between Japanese Americans and part-Japanese Americans. The top horizontal line demarcates separation between Okinawan Americans and part-Okinawan & part-mainland Japanese Americans and the bottom horiontal line demarcates separation between part-Okinawan & part-mainland Japanese Americans and mainland-Japanese Americans.**


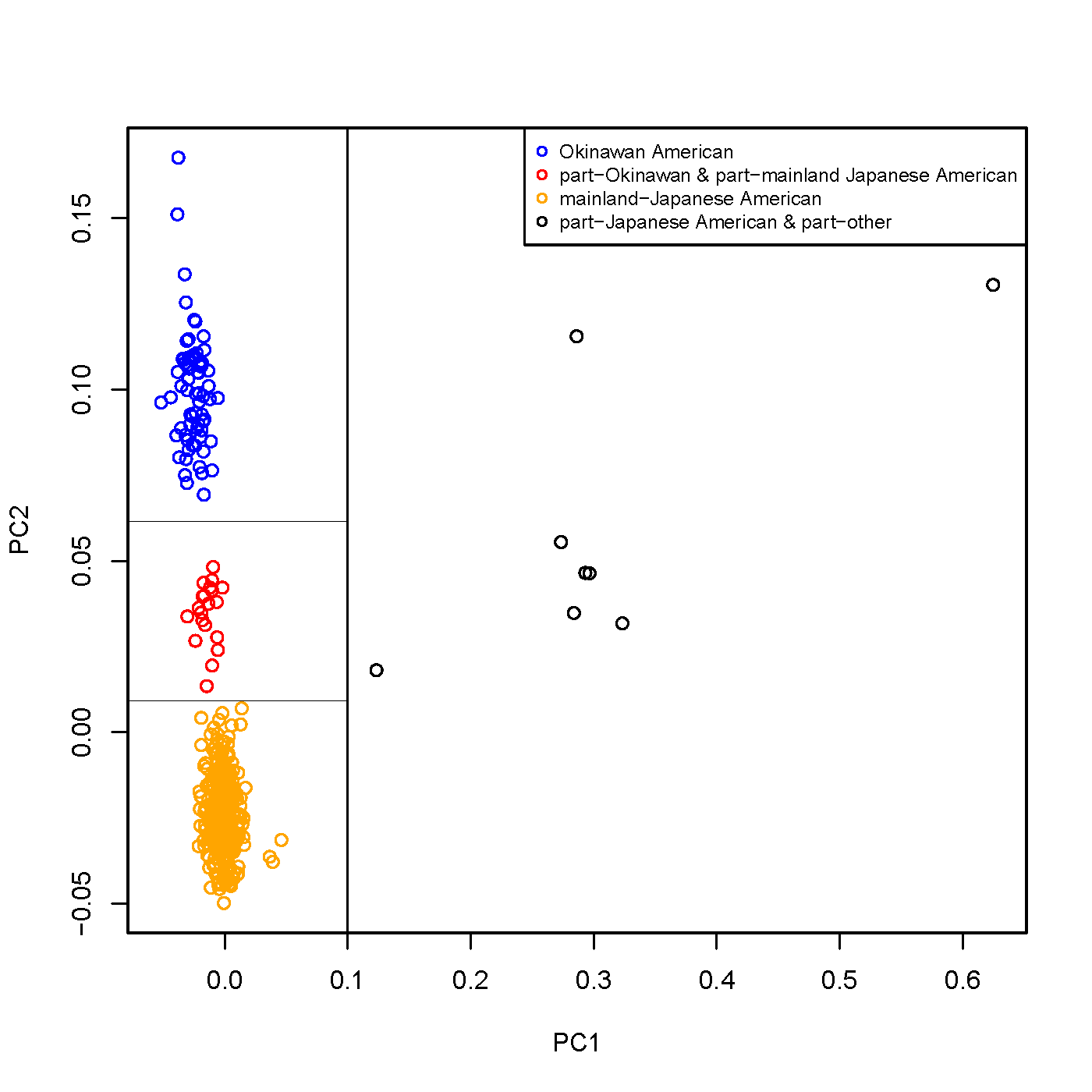

Supplement: S2 Fig — The vertical line demarcates separation between Japanese Americans and part-Japanese Americans. The top horizontal line demarcates separation between Okinawan Americans and part-Okinawan & part-mainland Japanese Americans and the bottom horiontal line demarcates separation between part-Okinawan & part-mainland Japanese Americans and mainland-Japanese Americans. (DOCX) [file pone.0279932.s002.docx]
